# ^18^F-FDG PET in the Diagnosis and Treatment of Primary Central Nervous System Lymphoma

**DOI:** 10.1155/2013/247152

**Published:** 2013-06-17

**Authors:** Nobuyuki Kawai, Keisuke Miyake, Yuka Yamamoto, Yoshihiro Nishiyama, Takashi Tamiya

**Affiliations:** ^1^Department of Neurological Surgery, Faculty of Medicine, Kagawa University, 1750-1 Miki-cho, Kita-gun, Kagawa 761-0793, Japan; ^2^Department of Radiology, Faculty of Medicine, Kagawa University, 1750-1 Miki-cho, Kita-gun, Kagawa 761-0793, Japan

## Abstract

This paper summarizes the usefulness and limitation of positron emission tomography (PET) with ^18^F-fluorodeoxyglucose (^18^F-FDG) in the diagnosis and treatment of primary central nervous system lymphoma (PCNSL). The ^18^F-FDG uptake in typical PCNSL is about 2.5 times higher than that in the normal gray matter, and the tumor can usually be identified visually. The ^18^F-FDG uptake pattern and value provide useful information for differentiating PCNSL from other enhancing malignant brain tumors especially glioblastoma (GB). The ^18^F-FDG uptake in typical PCNSL is usually homogenous, and the uptake value is significantly higher than that in GB. However, ^18^F-FDG PET often fails to show the presence of tumor in the brain as ^18^F-FDG uptake is faint in atypical PCNSL such as disseminated or nonenhancing lesions. ^18^F-FDG PET is also useful for evaluating the treatment response at a very early stage after the initial treatment. Pretreatment and posttreatment ^18^F-FDG uptake values may have a prognostic value in patients with PCNSL. In conclusion, ^18^F-FDG PET is very useful in the diagnosis of typical PCNSL and can differentiate PCNSL from other malignant brain tumors. However, the usefulness of ^18^F-FDG PET is limited in the diagnosis of atypical PCNSL.

## 1. Introduction

Although primary central nervous system lymphoma (PCNSL) is a rare tumor accounting for only 3–5% of all primary brain tumors, the incidence of PCNSL in developed countries is about 5 patients per 1 million person/year [1–3]. Epidemiological data have shown a continuous increase over the past three decades in the immunocompetent population, whereas the incidence seems to be decreasing in patients with acquired immunodeficiency syndrome (AIDS) since the development of highly active antiretroviral therapies [4]. PCNSL affects all age groups, with the peak incidence being in the fifth to seventh decades in non-AIDS patients. Therefore, the rising incidence of PCNSL may only represent the increasing age of the population. Recent studies have shown an encouraging improvement in the overall survival time when radiotherapy is combined with high-dose methotrexate- (MTX-) based chemotherapy [5, 6]. Young age and good Karnofsky performance score (KPS) at the time of diagnosis are reported to be associated with longer survival time [7]. Therefore, early diagnosis of PCNSL is essential to start early treatment before the patient's performance status has declined. Clinical diagnosis of PCNSL is sometimes difficult and delayed because common initial symptoms such as focal signs, raised intracranial pressure, and behavioral and personality changes especially in elderly patients are nonspecific [8]. Computerized tomography (CT) and magnetic resonance (MR) images in patients with PCNSL show single or multiple uniformly well-enhancing lesions that are usually located in the periventricular lesions and the basal ganglia and often involve the corpus callosum [8, 9]. These radiological findings are not pathognomonic for PCNSL and cannot accurately differentiate PCNSL from other tumorous or nontumorous brain lesions. Moreover, atypical MR findings such as disseminated lesions or no lesions are more prevalent in a recent study than formally reported in immunocompetent patients with PCNSL [10].

 Although CT and MR imaging are still the most important modalities in the diagnosis of PCNSL, modern metabolic imaging modalities other than conventional morphological imaging are increasingly used to improve accurate diagnosis of PCNSL. Positron emission tomography (PET) with glucose analogue ^18^F-fluorodeoxyglucose (^18^F-FDG) is one of the most attractive and widely used modalities for evaluating tumor metabolism noninvasively. Although PCNSL usually shows huge uptake of ^18^F-FDG, normally high uptake of ^18^F-FDG in the cerebral cortex, basal ganglia, and thalamus sometimes mask the presence of underlying PCNSL. Even in a rapid emerging clinical application, the role of ^18^F-FDG PET in PCNSL is not fully defined and reviewed systemically. This paper reviews the usefulness and limitation of ^18^F-FDG PET in the diagnosis and treatment of PCNSL.

## 2. Molecular Mechanism of ^18^F-FDG Uptake

The glucose analog ^18^F-FDG is a surrogate biomarker for glucose metabolism *in vivo* and is the most commonly used clinical PET radiotracer. The clinical applications of ^18^F-FDG PET continue to increase, especially in the field of oncology as ^18^F-FDG can be delivered from a hub-cyclotron center because of the relatively long half-life of ^18^F (110 min). The molecular mechanisms of ^18^F-FDG uptake in the cells were investigated intensively *in vitro* and *in vivo*. ^18^F-FDG enters the cells by the same membrane glucose transporter (GLUT) as glucose. More than 10 GLUTs have been identified to date; only GLUT-1 and GLUT-3 need to be considered in the normal and tumorous brain [11]. After passing the blood-brain barrier (BBB) via the GLUT, both ^18^F-FDG and glucose are phosphorylated by hexokinase. Unlike glucose-6-phosphate, ^18^F-FDG-6-phosphate is not a substrate of glucose-6-phosphate isomerase and does not undergo further metabolism in the glucose pathway and is trapped in the cells. As a result, the ^18^F-FDG uptake is a good reflection of glucose transport and phosphorylation by cells in the tumor. Several mechanisms have been shown to cause increased ^18^F-FDG uptake in malignant tumors including high cellular density, overexpression of GLUT [12–14], and increased hexokinase activity [13, 14]. ^18^F-FDG PET for tumor imaging is typically performed 45 to 60 minutes after an intravenous administration of ^18^F-FDG. This interval allows the increase in tumor tracer activity due to intracellular trapping of ^18^F-FDG-6-phosphate and the concomitant decrease in blood pool radiotracer and overall background tracer activity to improve the tumor-to-background ratio.

The degree of ^18^F-FDG uptake is measured to perform comparison within and between different patients and disease. The standardized uptake value (SUV) is a widely used method of measuring static ^18^F-FDG uptake in the lesion. The SUV is a semiquantitative value if all of the injected tracer is distributed evenly throughout the body and is computed as follows:

(1) $$\begin{array}{c} \text{SUV}=\frac{{\text{FDG}}_{\text{region}}}{\left({\text{FDG}}_{\text{dose}}/\text{WT}\right)}, \end{array}$$

where FDG_region_ is the decay-corrected regional radiotracer concentration in becquerel (Bq) per milliliter, FDG_dose_ is the administered ^18^F-FDG dose in Bq, and WT is the body weight in kilograms. Alternatively, the tumor-to-normal brain tissue (T/N) ratio is used for evaluating ^18^F-FDG uptake in the lesion. The T/N ratio is usually calculated by dividing the tumor SUV by the SUV value of the contralateral normal gray matter. The T/N ratio is not influenced by the injected radiotracer dose and the body weight, but the selection of normal brain tissue critically affects the calculated value.

## 3. ^18^F-FDG PET in the Diagnosis of PCNSL

### 3.1. Primary Diagnosis and Differentiation from Nontumorous Lesions

**Figure 1:**
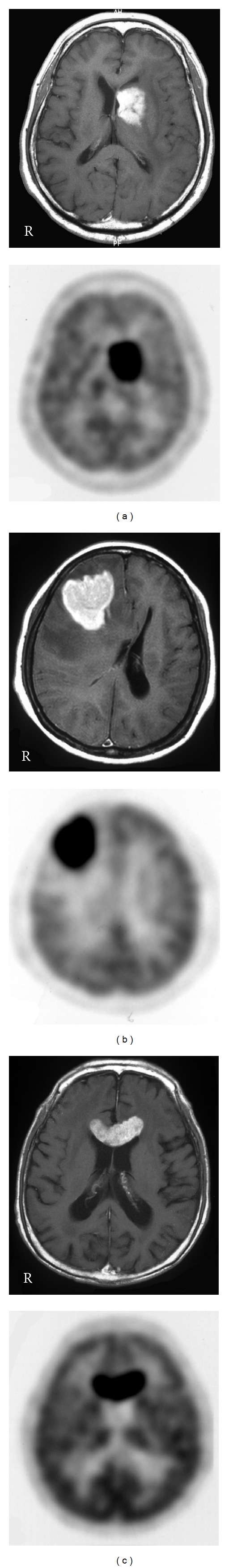
Contrast-enhanced T1-weighted MR (upper) and ^18^F-FDG PET (lower) images in PCNSL patients with typical MR findings. MR images show a homogenous enhanced lesion in the left basal ganglia (a), the right frontal white matter (b), and the corpus callosum (c). ^18^F-FDG PET images show a strong ^18^F-FDG uptake in the lesions.

**Figure 2:**
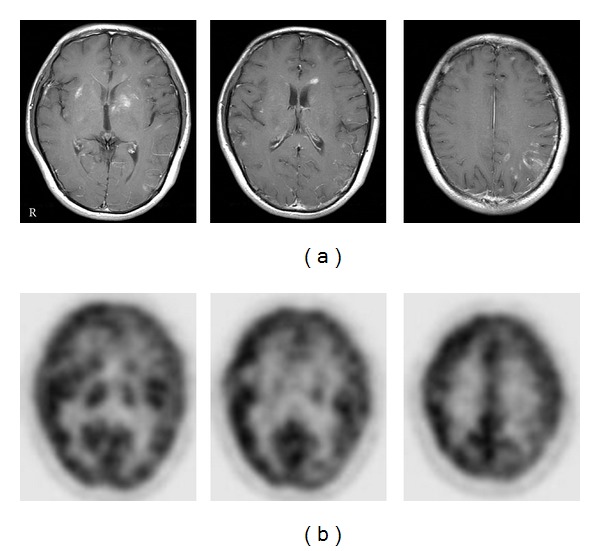
Contrast-enhanced T1-weighted MR (a) and ^18^F-FDG PET (b) images in an HIV-positive patient with toxoplasmosis. MR images show multiple, small, irregular enhanced lesions in the basal ganglia and the white matter. PET images show no ^18^F-FDG uptake in the lesions.

PCNSL has a very high cellular density and increased glucose metabolism and usually shows strong uptake of ^18^F-FDG in the tumor [15–20] (Figure 1). The semiquantitative ^18^F-FDG uptake values measured by maximum SUV (SUV_max⁡_) are reported to be 14–22 in PCNSL [15–18], and this value is about 2.5 times higher than the average SUV in the normal gray matter [15–17] (Table 1). In patients with acquired immunodeficiency syndrome (AIDS), ^18^F-FDG uptake in the lesions can be used to distinguish between human-immunodeficiency-virus-(HIV)-related brain disease such as cerebral toxoplasmosis and PCNSL [21–23] (Figure 2). The use of ^18^F-FDG PET in the diagnosis of PCNSL is not a new concept. In 1992, Rosenfeld et al. reported a strong ^18^F-FDG uptake in a group of 10 patients with PCNSL [19]. They also reported a patient who showed dramatic disappearance of ^18^F-FDG uptake in the tumor with steroid therapy [19]. Hustinx et al. examined SUVs in primary brain tumors on ^18^F-FDG PET and concluded that SUV measurements were influenced by a variety of factors, such as plasma glucose level, steroid treatment, tumor size and heterogeneity, time after injection, and previous radiation therapy [24]. Steroids have a cytotoxic effect in lymphoma cells and reduce ^18^F-FDG uptake in the tumor significantly causing false negative results of ^18^F-FDG PET in the diagnosis of PCNSL [15]. Moreover, nonspecific uptake of ^18^F-FDG has been reported in patients with nontumorous brain lesions such as intracerebral hematoma [25], brain abscess [26], and multiple sclerosis [27]. Animal studies have shown that inflammatory cells significantly contribute to ^18^F-FDG uptake in tumors. Kubota et al. reported that about 30% of ^18^F-FDG uptake was related to the non-tumorous tissue in a malignant tumor model in mice [28]. The extent of ^18^F-FDG uptake in the non-tumorous lesions depends on the increased density of inflammatory cells as well as disruption of the BBB in the lesion.

### 3.2. Differentiation from Other Malignant Brain Tumors

**Figure 3:**
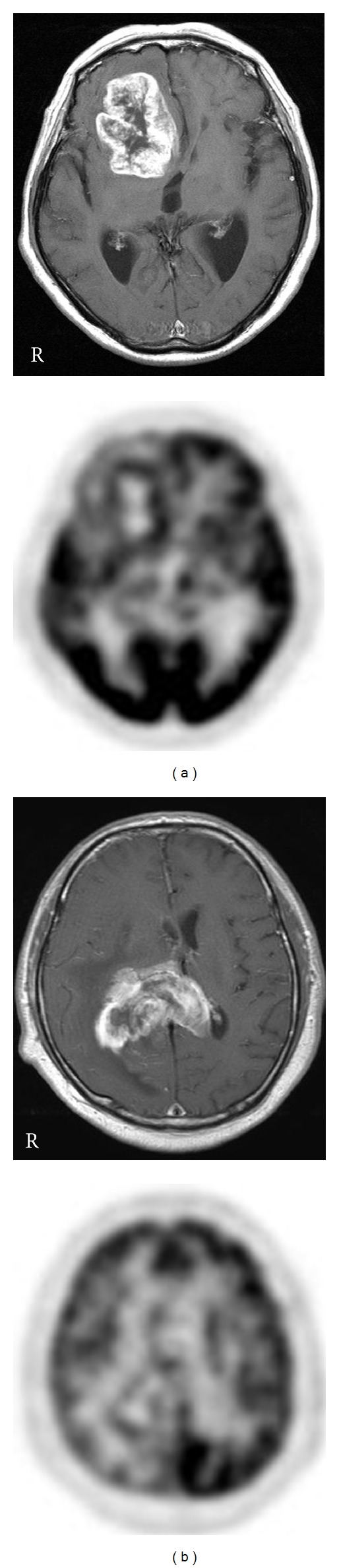
Contrast-enhanced T1-weighted MR (upper) and ^18^F-FDG PET (lower) images in GB patients. MR images show a heterogeneous ring-like enhanced lesion in the right frontal lobe (a) and the splenium (b). PET images show a mild ring-like ^18^F-FDG uptake in the lesions.

Recent studies have revealed that ^18^F-FDG PET can differentiate PCNSL from other malignant brain tumors such as glioblastoma (GB) and metastatic brain tumor [15, 18] (Figure 3). The ^18^F-FDG uptake in PCNSL is usually homogenous in contrast to inhomogeneous uptake in other malignant brain tumors. Kosaka et al. showed that metastatic brain tumors and GBs except for 1, case can be distinguished from PCNSL with ^18^F-FDG PET when the cutoff value was set at 15 of SUV_max⁡_ [15]. A recent study demonstrated the usefulness of ^18^F-FDG PET for differentiating between PCNSL and GB showing similar MR findings. ^18^F-FDG uptake in PCNSL (SUV_max⁡_ of 16.8 ± 7.2) was significantly higher than that in GB (SUV_max⁡_ of 8.2 ± 3.1; *P* < 0.01) [18]. The accuracy of ^18^F-FDG PET for lesion differentiation was 0.86 when the cutoff value was set at 12 of SUV_max⁡_ with a sensitivity of 100% and a specificity of 71.4% [18]. The overlying cortical gray matter sometimes shows glucose hypometabolism in PCNSL located in the deep white matter and the basal ganglia/thalamus. This finding is not a specific phenomenon in PCNSL and is reported in patients with gliomatosis cerebri due to disconnection of the cortical gray matter by tumor infiltration [29].

### 3.3. Diagnosis of Atypical PCNSL

**Figure 4:**
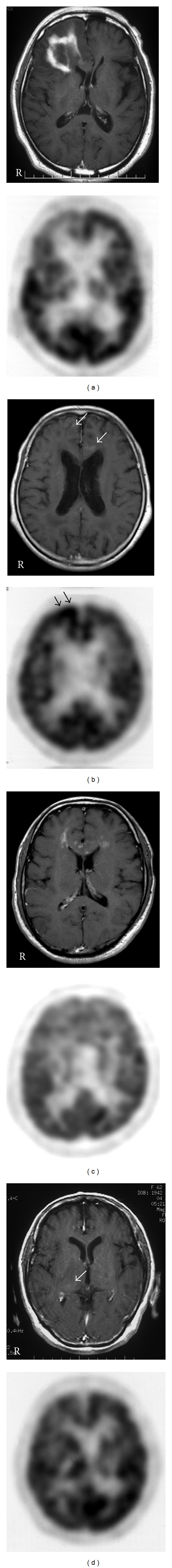
Contrast-enhanced T1-weighted MR (upper) and ^18^F-FDG PET (lower) images in PCNSL patients with atypical MR findings. MR images show a ring-like enhanced lesion in the right frontal lobe (a), multiple faint enhanced lesions in the right cerebral cortex and the corpus callosum (b), multiple small enhanced lesions in the bilateral frontal white matter and the corpus callosum (c), and no enhancing lesion in the brain (d). PET images show no ^18^F-FDG uptake in the lesions except for moderate ^18^F-FDG uptake in the right frontal cortex (arrow) in case (b).

PCNSL with typical radiological findings shows strong ^18^F-FDG uptake in almost all cases, and ^18^F-FDG PET provides valuable information in the primary diagnosis of PCNSL. However, PCNSL sometimes demonstrates atypical radiological findings such as disseminated or nonenhancing lesions (no lesions) in contrast-enhanced MR or CT images [9, 10]. Such atypical findings in non-AIDS patients with PCNSL were more prevalent in a recent study, showing that 13% of the patients had no lesions and 7% of the patients had disseminated lesions [10]. Ring-like enhancement occurs in more than 50% of the lesions in AIDS-related PCNSL, but also in 6–13% of the lesions in non-AIDS PCNSL [9, 10]. ^18^F-FDG uptake in PCNSL with atypical radiological findings is not increased sufficiently to detect the tumor visually because of normally high background uptake of ^18^F-FDG in the brain (Figure 4). Kawai et al. revealed that 3 of the 4 ^18^F-FDG PET failed to show the presence of PCNSL with atypical radiological findings visually [30]. Therefore, ^18^F-FDG PET is not a perfect tool, and caution is necessary especially in the diagnosis of atypical PCNSL. To date no imaging modality can definitively diagnose PCNSL, and early tumor biopsy is still recommended when PCNSL is suspected especially with atypical radiological findings [31].

### 3.4. Detection of Occult Systemic Lymphoma

PCNSL is, by definition, a non-Hodgkin's lymphoma restricted to the CNS. Standard staging for PCNSL needs to examine contrast-enhanced CT of the chest, abdomen, and pelvis and sometimes bone marrow biopsy to exclude systemic lymphoma [32]. Conventional body staging in patients initially diagnosed with PCNSL showed an occult systemic lymphoma in about 4% of the patients [33]. The clinical significance of identifying systemic disease is uncertain, but at least 5% of PCNSL relapse outside the CNS [34]. ^18^F-FDG PET may be more sensitive than conventional body staging and may disclose higher rates of concomitant systemic disease at initial PCNSL diagnosis. Mohile et al. demonstrated that 7% of patients initially diagnosed PCNSL were found to have systemic lymphoma by staging ^18^F-FDG whole body PET scan even when body CT scans and bone marrow biopsies were negative [35]. Detection of systemic lymphoma at the time of initial PCNSL diagnosis may play important roles regarding the origin of the disease and treatment strategies. 

*Summary*

- ^18^F-FDG uptake value in PCNSL is about 2.5 times higher than that in the normal gray matter, and the tumor can usually be identified in the brain visually.
- Steroid treatment significantly reduces ^18^F-FDG uptake in the tumor and can cause false negative results of ^18^F-FDG PET in the diagnosis of PCNSL.
- ^18^F-FDG PET can differentiate PCNSL from other malignant brain tumors such as GB and metastatic brain tumor with high sensitivity.
- ^18^F-FDG uptake in PCNSL with atypical radiological findings such as disseminated or nonenhancing lesions is not increased sufficiently to detect the tumor visually compared to the surrounding brain.
- Conventional body scan with ^18^F-FDG PET in patients initially diagnosed as PCNSL is occasionally useful to detect occult systemic lymphoma.

## 4. ^18^F-FDG PET in the Treatment of PCNSL

### 4.1. Early Treatment Response

**Figure 5:**
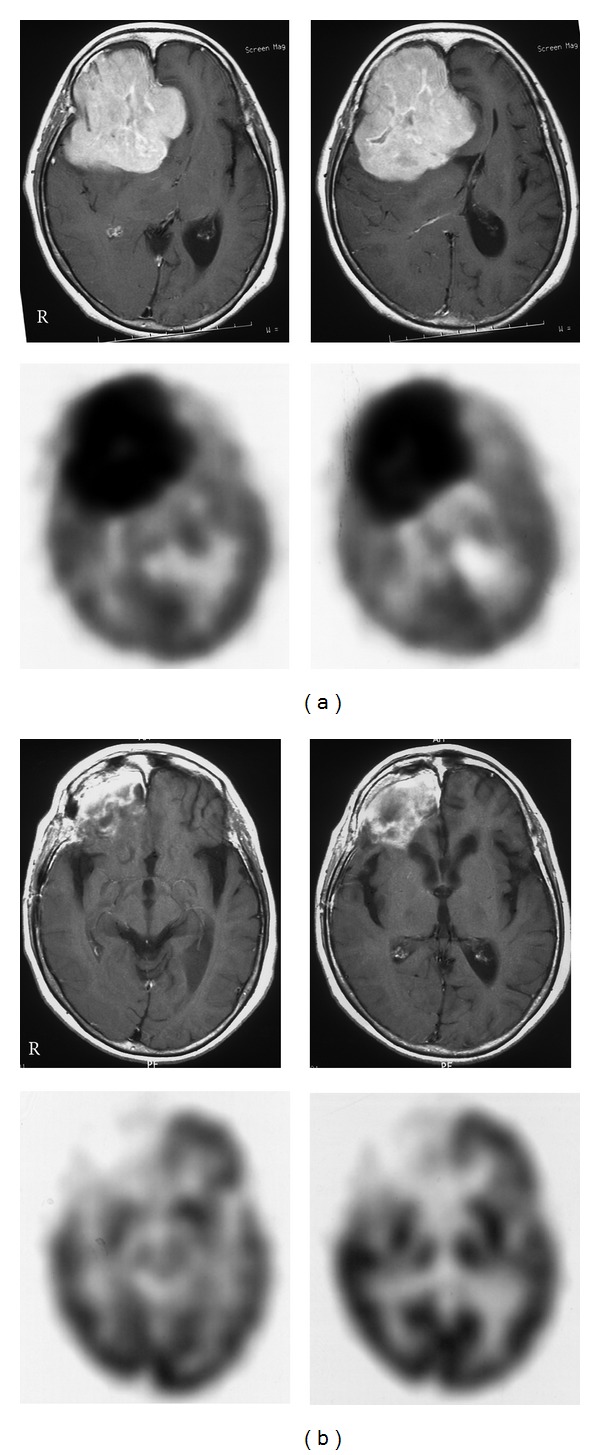
Contrast-enhanced T1-weighted MR (upper) and ^18^F-FDG PET (lower) images in a patient with PCNSL in the right frontal lobe before (a) and after (b) the first cycle of chemotherapy. MR images show a well-enhanced large mass lesion in the right frontal lobe, and PET images show a huge ^18^F-FDG uptake in the lesion before treatment (a). After the first chemotherapy, MR images show a residual enhanced lesion in the right frontal lobe; however, PET images show no increased ^18^F-FDG uptake in the lesion (b).

PCNSL is one of the most treatment-responsive malignant tumors in the brain. In recent years, high-dose MTX-based chemotherapy before radiotherapy has significantly extended the survival time compared to conventional chemotherapy with cyclophosphamide, adriamycin, vincristine, and prednisone (CHOP)-based regimens given either before or after radiotherapy [5, 6]. However, it is reported that about 10–35% of tumors are refractory to the high dose MTX-based regimen, and up to 60% of complete responders show tumor recurrence during follow-up [36]. Early evaluation of the initial treatment response is very important because salvage treatment may improve the outcome and quality of life [37]. Although MR imaging is the standard method for evaluating the treatment response in PCNSL [32], few studies have addressed the question of whether early tumor response, according to MRI criteria, in patients still under therapy helps to predict the long-term outcome in PCNSL [38]. In systemic lymphoma patients, an early quantitative measurement of metabolic response with ^18^F-FDG PET was reported to provide more valuable prognostic information than conventional modalities [39]. Changes in metabolic imaging with ^18^F-FDG PET occur soon after the initiation of therapy. Palmedo et al. studied 8 PCNSL patients with ^18^F-FDG PET after completion of chemotherapy or after the first cycle of chemotherapy, and the results were compared with the follow-up examinations [20]. They showed that ^18^F-FDG PET was able to predict complete remission or to diagnose tumor recurrence after chemotherapy in all patients [20]. Kawai et al. demonstrated that ^18^F-FDG PET examined within 3 weeks of the first chemotherapy showed a significant decrease of ^18^F-FDG uptake in the tumor compared with that before treatment [40]. The reduction of ^18^F-FDG uptake significantly correlated with the decrease of tumor size on the follow-up MR images [40]. These results indicate that metabolic imaging with ^18^F-FDG PET can be used to accurately evaluate treatment response at a very early stage, sometimes preceding changes on MRI (Figure 5). Early therapeutic monitoring might have an impact on deciding whether the treatment regimen should be maintained or changed. If patients with a poor early response were identified, then modification could be taken at an early stage, before many more cycles of ineffective therapy were delivered. Again, caution is necessary in interrupting ^18^F-FDG PET images especially after treatment because ^18^F-FDG uptake in the tumor is not solely due to tumor cell metabolism but it also due to uptake in stromal and inflammatory cells [28]. 

### 4.2. Prognostic Considerations

A recent study showed that pretreatment ^18^F-FDG uptake may have a prognostic value in newly diagnosed PCNSL. The overall survival time of patients with low to moderate ^18^F-FDG uptake (SUV_max⁡_ < 12) was significantly longer than that of patients with high ^18^F-FDG uptake (SUV_max⁡_ ≥ 12) [16]. PCNSL with high ^18^F-FDG uptake tended to exhibit poor treatment response compared to that with low to moderate ^18^F-FDG uptake [16]. The ^18^F-FDG uptake value may represent tumor aggressiveness in PCNSL. Further clinical trials are needed to define the best way to utilize ^18^F-FDG PET information in designing true response-adapted therapies and to improve outcome in patients with PCNSL. 

*Summary*

- ^18^F-FDG PET can be used to evaluate treatment response of PCNSL at a very early stage after treatment.
- ^18^F-FDG PET is able to predict complete remission or to diagnose tumor recurrence of PCNSL after treatment.
- Pretreatment ^18^F-FDG uptake may have a prognostic value in newly diagnosed PCNSL.

## 5. Conclusions

The application of ^18^F-FDG PET is currently increasing in clinical neurooncology. This review summarizes the usefulness and limitation of ^18^F-FDG PET in the diagnosis and treatment of PCNSL. ^18^F-FDG PET is very useful in the diagnosis of typical PCNSL, usually showing strong uptake of ^18^F-FDG in the tumor. The uptake value is about 2.5 times higher than that in the normal gray matter, and the tumors can be identified in the brain visually. The ^18^F-FDG uptake pattern and value provide useful information to differentiate PCNSL from other enhancing malignant brain tumors especially GB. However, the usefulness of ^18^F-FDG PET is limited in the diagnosis of PCNSL with atypical radiological findings. ^18^F-FDG PET is also useful for evaluating the treatment response after initial chemotherapy and determining the strategy at a very early stage. Pretreatment and posttreatment ^18^F-FDG uptake values may have a prognostic value in patients with PCNSL. In a modern metabolic imaging era, ^18^F-FDG PET is useful when differential diagnosis of brain tumors is difficult, and PCNSL is considered as one of the differential diagnoses, but ^18^F-FDG PET is not a perfect tool, and early tumor biopsy is still necessary especially with atypical radiological findings.

## Figures and Tables

**Table 1:** Literature review: ^18^F-FDG SUV_max⁡_ and T/N ratio in PCNSL.

Study	*n*	Age (range)	SUV_max⁡_	T/N ratio
Kosaka et al. (2008) [15]	7^1^	—	22.2 ± 5.0	2.31 ± 0.70
Kawai et al. (2010) [16]	17^2^	65 (47–79)	13.5 ± 5.4	2.54
Kawase et al. (2011) [17]	13^2^	70 (54–80)	13.9 ± 5.7	2.74 ± 1.25
Makino et al. (2011) [18]	14	—	16.8 ± 7.2	—

^1^Two patients were treated with dexamethasone before PET study.

^2^Seven patients are overlapped (same institution).
